# Interventional treatment of obesity and diabetes: An interim report on gastric electrical stimulation

**DOI:** 10.1007/s11154-016-9350-7

**Published:** 2016-04-23

**Authors:** Harold E. Lebovitz

**Affiliations:** State University of New York Health Science Center at Brooklyn, 450 Clarkson Avenue, Box 1205, Brooklyn, New York, 11203 USA

**Keywords:** Gastric electrical stimulation, Obesity, Type 2 diabetes, Weight loss, Glycemic control

## Abstract

Gastric electrical stimulation has been applied to treat human obesity since 1995. Dilatation of the stomach causes a series of neural reflexes which result in satiation and satiety. In non-obese individuals food ingestion is limited in part by this mechanism. In obese individuals, satiation and satiety are defective and unable to limit energy intake and prevent excessive weight gain. Several gastric electrical stimulatory (GES) devices have been developed, tested in clinical trials and even approved for the treatment of obesity. The design and clinical utility of three devices (Transend®, Maestro® and DIAMOND®) that have been extensively studied are presented as well as that of a new device (abiliti®) which is in early development. The Transcend®, a low energy GES device, showed promising results in open label studies but failed to show a difference from placebo in decreasing weight in obese subjects. The results of the clinical trials in treating obese subjects with the Maestro®, a vagal nerve stimulator, were sufficient to gain approval for marketing the device. The DIAMOND®, a multi-electrode GES device, has been used to treat type 2 diabetes and an associated benefit is to reduce body weight and lower systolic blood pressure.

## Introduction

Energy balance and body weight are regulated by complex interactions between brain, gastrointestinal tract, and adipose tissue [1–3]. The brain contains neuronal centers that regulate appetite and hunger (hypothalamus), receives signals from the periphery which indicate its nutritional status (area postrema and nucleus tractus solitarius of the hindbrain) and assesses the state of satisfaction and reward behavior (hippocampus and amygdala) [3–6]. The gastrointestinal tract sends both neural signals through the vagus and sympathetic nerves and circulating hormones which inform the hindbrain centers of the nutritional intake and the metabolic state of the periphery [3–7]. These signals are sent forward to the hypothalamus, hippocampus and amygdala where they are integrated and appropriate responses for feeding and secretion of pancreatic islet hormone are modulated [3–6]. The adipose tissue sends circulating hormones (leptin) and nutrients (free fatty acids) to the hypothalamic centers. The signals from the gastrointestinal tract and adipose tissue influence whether the activity of the median eminence orexigenic neuropeptide Y (NPY)/agouti-related peptide (AgRP) neurons or anorexigenic pro-opiomelanocortin (PMOC) neurons predominates [1–6].

The gastrointestinal tract has a dense submucosal neural plexus which is activated by stretch and/or ingested nutrients [7–9]. Additionally, the gastrointestinal tract secretes hormones such as ghrelin, glucose independent polypeptide (GIP), glucagon-like peptide 1 (GLP-1), peptide YY (PYY), oxyntomodulin, and cholecystokinin which can act on peripheral tissues such as pancreatic islets to regulate insulin, glucagon and pancreatic polypeptide secretion or directly on areas in the brain such as the median eminence and area postrema in which the blood brain barrier is minimal [1–4].

Such a complex regulatory system provides many potential abnormalities which could cause disturbances of energy balance [9, 10] such as obesity and/or diabetes. It likewise presents the potential for multiple different strategies for treating obesity and/or diabetes mellitus. However, a significant challenge in treating obesity is that modifying one component of the system results in a compensatory adaptation in other components and negates the benefit of the intervention.

The stomach is a key component of this energy regulatory system. It senses the quantity and quality of the nutrient intake and through ghrelin secretion influences orexigenic activity [3, 4], through its neural input into the hindbrain influences satiety, satiation and islet hormone secretions [9, 10] and through its regulation of nutrient entry into the intestine the secretion of gastrointestinal hormones [3, 4]. It is little wonder that many interventions to treat obesity and/or diabetes mellitus center around the stomach. Most effective bariatric surgery interventions such as gastric banding, sleeve gastrectomy and gastric by-pass alter gastric function as an important component of their mechanism of action. Interventional strategies other than surgical alterations of the stomach such as balloon insertion or electrical stimulation [11, 12] have been evaluated for their effects on obesity and/or diabetes mellitus. The purpose of this review is to provide an interim update on the results of gastric electrical stimulation as a treatment for obesity and type 2 diabetes mellitus.

## History of gastric electrical stimulation

The stomach has an intrinsic electrical pacemaker which regulates its slow wave contractions and its motor activity. These factors are coordinated in a specific physiologic pattern by food ingestion. Disturbances in the neural control of the stomach leads to uncoordinated motility (gastroparesis) which is one of the chronic complication of poor glycemic control in patients with diabetes mellitus [13]. The first efforts in applying external electric signals to the stomach were to treat the abnormal neuromuscular activity associated with gastroparesis [11, 12]. Gastric pacing has been applied to reverse the dysrhythmic activity and delayed gastric emptying [14]. Different patterns of electrical pacing are used to treat gastroparesis with variable degrees of success [11, 12, 14, 15]. Gastric electrical stimulation for the treatment of obesity began in the early 1990s with the demonstration that it could reduce food intake and weight gain in pigs. By 1995 gastric stimulation to treat human obesity began. Several different types of electrical stimulation have been extensively used to treat obesity and/or diabetes in humans [11, 12, 15, 16] and they are the subject of this update. It is important in evaluating these procedures to evaluate the design of the clinical studies, whether the studies included blinded “placebo” controls and the significance of the pre-specified end points. Surgical studies frequently record weight loss as percent decrease in excess body weight rather than percent absolute weight loss. Excess body weight is defined as the weight above the midpoint of the 1983 Metropolitan Life Insurance tables for a given height and gender. Excess body weight loss divided in half is usually equivalent to percent decrease in body weight.

### Low energy GES device-transcend® stimulator

The Transcend® Implantable Gastric Stimulator originally manufactured by Transneuronix (Mt. Arlington, NJ) and subsequently purchased by Medtronic Inc. (Elizabeth, NJ) is a gastric pacemaker implanted laparoscopically below the pes anserinus 3 cm from the edge of the lesser curvature and 6 cm from the pyloris [15, 16]. The parameters of the electrical signal are: amplitude 10 mA, pulse width 208 μs, frequency 40 Hz. The pulse is on 2 s and off 3 s for 24 h each day. Open label, primarily uncontrolled studies in approximately several hundred morbidly obese subjects treated for 6 months to 2 years in various European centers found a mean decrease in excess body weight of approximately 20 to 30 % [16]. A randomized, placebo controlled trial (0–01) of 103 obese patients in 10 centers in US with a duration up to 29 months failed to show much benefit in reducing excess body weight [14]. The definitive study with the Transcend device was the SHAPE clinical trial [17]. Patients 18 to 65 years with a BMI of 35 to 55 kg/m^2^were prescreened according to the BaroScreen screening algorithm (Medtronic, Minneapolis, MN). Only candidates with a predicted percent excess weight loss ≥15 % within 12 months were considered for enrollment. Prospective patients were then further screened by a psychologist or dietician. Ultimately 190 of 4802 candidates were enrolled in the study. All patients were implanted with the device and all were instructed to consume a diet with a 500 cal deficit. Randomization to device on or device off occurred 2 weeks after surgery. Patients were followed monthly for 12 months. At the end of 12 months there was no difference in excess weight loss between the treatment and control groups (−12.2 ± 17.4 vs. -11.9 ± 17.1 %).

### Vagal blockade

Vagotomy has been reported to cause early satiation associated with decreased gastric accommodation and delayed gastric emptying, short term loss of appetite, weight loss, and pancreatic exocrine deficiency. These effects lessen with time as a result of the formation of collateral innervations and up regulation of metabolic or neural pathways. Based on those observations that interruption of vagal impulses may cause weight loss, a device was developed to induce intermittent intra-abdominal vagal nerve blockade to treat obesity using high-frequency electrical currents [18]. The Maestro ™ Device (EnteroMedics Inc) consists of one electrode attached to each trunk of the vagus nerve, a neuroregulator placed subcutaneously and an external programmer [18, 19]. The electrodes are implanted laparoscopically around the anterior and posterior branches of the vagus nerve at the level of the esophageal-gastric junction. The leads are connected to the neuroregulator which is implanted in a midline subcutaneous pocket just below the Xiphoid process. The external controller delivers an electrical current with a frequency of 5000 Hz, and an amplitude of 6 mA. The device is turned on in the morning and turned off at night. The current is administered alternating 5 min on and 5 min off for 12 h daily. The mean excess weight loss in a pilot study of 31 morbidly obese subjects treated for 6 months was 14.2 % [18]. Sub studies in a few patients showed a 30 % decrease in caloric intake and a significant decrease in pancreatic polypeptide secretion.

Several large randomized controlled trials have assessed the results of long-term treatment of the vagal blockade device on weight loss in morbidly obese individuals. The EMPOWER study recruited 294 subjects with a mean BMI 41 ± 1 kg/m^2^, mean age 46 ± 1 years and 90 % women [19]. The patients were randomized 2 to 1 to an active controller (5000 Hz, 3 to 6 mA, duty cycle alternating 5 min on and 5 min off) and a placebo controller (during the on cycle two bursts of 13 impulses at 1000 Hz and 3 mA of 26 ms duration at time 0 and 3 min and 40 HZ and 1 mA thorough the remainder of the on cycle). The duration of the study was 12 months and the endpoint was percent loss of excess weight. Patients activated the device a minimum of 9 h and a maximum of 16 h daily. All subjects received 15 individual counseling sessions on weight management. There was no difference between percent excess weight loss in the treated versus control patients at 12 months (17 ± 2 % versus 16 ± 2 %).

Several obvious flaws occurred in the design and implementation of the EMPOWER study which could have resulted in the failure to demonstrate a significant effect of the vagal blockade. Two significant problems were the control group actually received a lesser rather than no stimulation and the time of daily stimulation in both groups was varied by the patients and was not constant. An additional 12 month, randomized, blinded, controlled study was carried out in 239 type 2 (BMI 35–39.9 kg/m^2^) and type 3 (BMI ≥ 40 kg/m^2^) obese patients in Australia and US [20]. They were randomized 2:1 to an active and a placebo treatment. The placebo treatment included laparoscopic incisions and implantation of the neurotransmitter. However the electrodes were not implanted around the vagus nerve trunks and the electrical signal was dissipated within the neurotransmitter [18]. Additionally, all patients were stimulated for 12 h daily. As in the previous study, all patients received individualized dietary counseling sessions. Though the mean difference in excessive weight loss between the treatment and control groups at 12 months failed to meet the pre-specified primary endpoint difference margin of 10 %, the difference was statistically significant (24.4 % versus 15.9 %, *p* = 0.002). The difference in body weight of 3.2 % though statistically different was hardly impressive. In a 6 month unblinded extension the Vbloc treated patients maintained their weight loss while the placebo group regained much of the weight that they had lost [21]. Adverse effects more frequent in Vbloc patients were heartburn, dyspepsia and abdominal pain.

A pilot open label study of Vbloc treatment of obese patients with type 2 diabetes was carried out over a period of 24 months [22]. The baseline characteristics of the patients were: women 61 %, mean BMI 37 ± 3 kg/m^2^, mean body weight 107 ± 16 kg, mean HbA1c 7.8 %; 62 mmol/mol (range 7.4 to 8.1 %; 57 to 66 mmol/mol). The patients were on oral antidiabetic medications. The patients were seen weekly for month 1, biweekly through month 3, then monthly through year 1 and bimonthly through year 2. At each visit the patients received individual weight management counseling. Twenty three patients completed the 24 months and the mean percent decrease in body weight was 6.9 % (*p* = 0.0001), in HbA1c 0.6 %; 5 mmol/mol (*p* = 0.0026), in mean arterial blood pressure 2 mmHg (*p* = 0.48) and in waist circumference 7 cm (*p* = 0.0001). Adverse events includes heartburn (29 %), constipation (21 %), pain at the neuroregulator site (18 %) and nausea (11 %). Two surgical revisions and 2 explants were done during the 24 months. It is not possible to determine the extent to which the outcomes were due to vagal blockade or to the weight management program as there was no control group with the weight management program either with or without a placebo device.

### Multi-electrode non-excitatory prandial gastric electrical stimulation

The Tantalus gastric electrical stimulatory device (DIAMOND ™) is based on different principles than the other gastric electrical stimulatory devices [23]. It consists of three pairs of electrodes implanted under laparoscopic conditions into the fundal, anterior and posterior antral regions of the stomach. The electrodes are attached to a pulse generator which is placed in a pocket created by the surgeon in the abdominal subcutaneous fat. The pulse generator is attached to a charging coil. The characteristics of the electric pulse generated is set by an external programmer. The device detects food ingestion by a change in impedance of the fundal electrodes as the fundus dilates and by a decrease in electrical slow wave activity detected by the antral electrodes. When the food detection signals are received by the pulse generator, it sends electrical pulses to the antral electrodes timed to arrive during the refractory period of the intrinsic electrical signal. A pulse received during the refractory period does not change the rate of antral contraction, but does increase the force of contraction 2 to 4 fold and increases the neural signals transmitted from the antrum to the hindbrain. The battery in the pulse generator is recharged weekly by placing the charger which is connected to an external electric source over the charging coil of the impulse generator. Gastric contractility modulation is delivered for 75 min starting at meal detection. The electrical impulses is delivered to the antral electrodes synchronized to the local intrinsic gastric slow wave. Electrical pulses use a biphasic symmetric waveform having a phase duration of 6 ms, a repetition rate of 83 Hz, a pulse duration of 1200 ms and an amplitude of 5 to 10 mA depending on the patient. The pulse is applied 15 min on and 15 min off for the 75 min of activation.

Several large clinical trials have assessed the effects of the DIAMOND device in treating obese patients with type 2 diabetes inadequately controlled on one or more oral antidiabetic agents (metformin either alone or combined with a sulfonylurea or a thiazolidinedione) [23, 24]. Inclusion criteria for the patients recruited were: age 25 to 70 years, BMI > 25 kg/m^2^, HbA1c >7.0; 53 mmol/mol to <10.5 %; 91 mmol/mol, Patients enrolled in the studies were instructed to continue their current lifestyle and no specific attempts were made to enforce any specific life style alterations. After screening and baseline laboratory studies, the patients were implanted and one week following implantation, the DIAMOND device was programmed for the appropriate electrical signal generation. The salient finding in all the studies was a significant improvement in HbA1c, a decrease in body weight and a decrease in systolic blood pressure. Table 1 provides a summary of the results of open label DIAMOND treatment in these inadequately controlled obese patients with type 2 diabetes. The mean HbA1c decreased from a baseline value of 8.38 %; 68 mmol/mol to 7.44 %; 58 mmol/mol after 12 months of treatment. The mean weight decreased from 107.1 kg to 102.5 kg. During the clinical trials, it was observed that there was significant patient variability in the response to the DIAMOND treatment. A well-recognized cause of variability in HbA1c response to medical therapies is the influence of baseline HbA1c levels. A retrospective analysis of the data confirmed that the decrease in HbA1c in response to DIAMOND treatment was a function of the baseline HbA1c level, but another independent factor predicting the glycemic response was whether the fasting plasma triglyceride level was in the normal range (≤1.7 mmol/l) or was elevated (> 1.7 mmol/l). Table 1 illustrates the effect of the fasting plasma triglyceride on the response to DIAMOND treatment. Patients with normal fasting plasma triglyceride levels had twice as great a decrease in HbA1c and body weight as those with elevated fasting plasma triglyceride levels. The mechanism of this ±triglyceride effect is likely due to an interaction on the gut-brain-metabolic regulatory pathway. A one year pilot study in obese Chinese patients with type 2 diabetes comparing DIAMOND treatment to insulin treatment [25] showed that DIAMOND treatment was superior because it decreased body weight, reduced body fat and waist circumference and lowered systolic blood pressure (Table 2).

**Table 1:** Effect of 12 months DIAMOND treatment in European/US Patients with type 2 diabetes inadequately treated with oral antihyperglycemic agents [23, 24]

Population	Number	HbA1c Baseline (%; mmol/mol)	HbA1c 12 months (%; mmol/mol)	Decrease in HbA1c at 12 months (%; mmol/mol)	Body weight Baseline (kg)	Body weight at 12 months (kg)	Decrease in body weight at 12 months (kg)
All Patients	76	8.38 ± 0.09 68 ± 0.73	7.44 ± 0.11 58 ± 0.86	−0.94 ± 0.13** -10 ± 1.39	107.1 ± 2.53	102.5 ± 2.55	−4.6 ± 0.85*
Normal Triglycerides (≤1.7 mmol/l)	43	8.44 ± 0.13 69 ± 1.06		−1.23 ± 0.19 -14 ± 2.16	104.3 ± 3.35		−6.00 ± 1.34
High Triglycerides (> 1.7 mmol/l)	33	8.30 ± 0.13 67 ± 1.05		−0.62 ± 0.16 -7 ± 1.81	110.6 ± 3.32		−2.65 ± 0.78

*p < 0.001

**p < 0.00001

**Table 2:** The effect of DIAMOND treatment versus insulin treatment on metabolic parameters in obese Chinese patients with type 2 diabetes inadequately controlled on oral antihyperglycemic agents. Data are the change from baseline to 12 months [25]

Metabolic Parameter	DIAMOND treatment (*N* = 8)		Insulin Treatment (*N* = 8)		Difference between DIAMOND and Insulin treatment (*p* value)
	Baseline	Change at 12 months	Baseline	Change at 12 months	
Body weight (kg)	80.4	↓3.2	86.0	↑2.4	< 0.01
BMI (kg/m^2^)	29.4		30.8		
Body fat (%)	33.5	↓3.2	28.0	↑1.5	0.002
Waist Circumference (cm)	98.9	↓3.9	104.5	↑1.5	0.002
Mean systolic blood pressure (mm Hg	129.0	↓4.5	130.6	↑2.3	0.038
HbA1c (%; mmol/mol)	9.1; 76	↓0.9; 10	8.9; 74	↓0.3 ;4	0.46
Normal TG (3)		↓2.6; 28			
High TG (5)		↓0.4; 4			

A fundamental issue with all electrical stimulatory devices is the proof that the result measured is a true effect of the electrical signal and not a placebo effect due to the surgical intervention, the presence of an implanted device or some lifestyle modification. The proof requires a randomized, placebo- controlled trial that demonstrates that the effect generated by the signal is statistically greater than that caused by the placebo conditions. The DIAMOND is a device to treat diabetes and improve glycemic control. Its effects in decreasing weight and lowering systolic blood pressure are added benefits, Evidence that the DIAMOND electrical signals cause the improved glycemic control in patients with type 2 diabetes was shown in a 48 week, blinded, cross-over study which compared the effects of the implanted device turned on to the implanted device turned off on HbA1c levels in inadequately controlled patients with type 2 diabetes [26]. The change in HbA1c from baseline to 48 weeks in which the last 24 weeks had the device turned off was not significant (8.32 ± 0.16 %; 67 ± 1.29 mmol/mol to 8.06 ± 0.32 %; 65 ± 2.68 mmol/mol, *p* = 0.46). In contrast the change in HbA1c from baseline to 48 weeks in which the electrical signal was turned on for the last 24 weeks was significantly decreased (8.40 ± 0.15 %; 68 ± 1.21 mmol/mol to 7.47 ± 0.15 %; 58 ± 1.16 mmol/mol, *p* = 0.001).

**Fig. 1:**
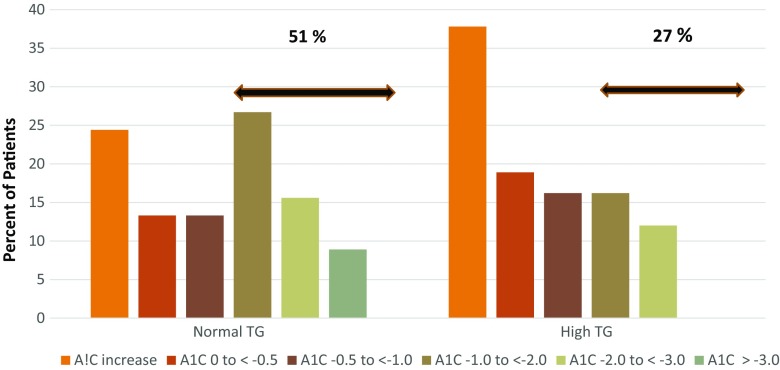
Frequency of HbA1c change in patients with type 2 diabetes with normal (≤ 1.7 mmol/l) and high (> 1.7 mmol/l) fasting plasma triglyceride (TG) levels treated with meal-mediated gastric electrical stimulation for 12 months. Mean baseline HbA1c for 45 normal TG patients 8.43 ± 0.12 %; 69 ± 0.98 mmol/mol and for 37 high triglyceride patients 8.22 ± 0.12 %; 66 ± 0.96 mmol/mol. Fifty one % of normal TG patients and 27 % of high triglyceride patients had a decrease in HbA1c ≥1 %. Conversion of HbA1c from % to IFCC standard mmol/mol; 5.0 % = 31 mmol/mol and for each additional 1 % add 11 mmol/mol

**Fig. 2:**
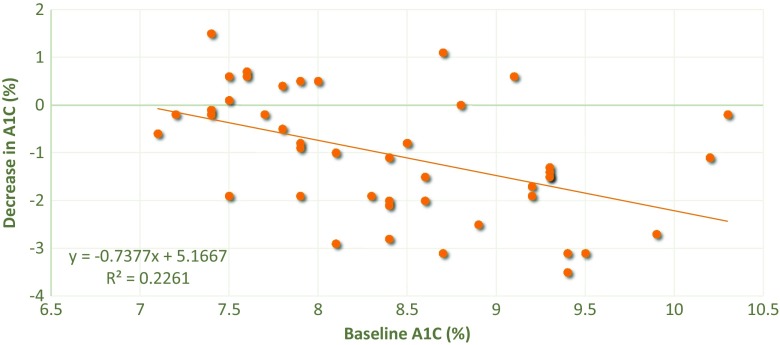
Decrease in HbA1c as a function of their baseline HbA1c in 45 normal fasting plasma triglyceride obese type 2 diabetic patients treated with meal-mediated gastric electrical stimulation for 12 months. Correlation Coefficient is 0.475, *p* = 0.0001. Conversion HbA1c from % to IFCC standard mmol/mol: Each 0.5 % =5.5 mmol/mol

The metabolic effects of DIAMOND’s electrical stimulatory activity are complex and mediated through a gut-brain-liver & pancreatic islet regulatory pathway. It is therefore not unexpected that there is significant differences in individual responses to the DIAMOND device in obese patients with type 2 diabetes. Enriching the treatment population with patients with normal fasting plasma triglycerides and higher baseline HbA1c increases the probability of a good response to treatment but there are clearly additional factors which influence response. Figure 1 plots the distribution of the magnitude of HbA1c decrease seen in 45 treated patients with normal fasting triglycerides. Fifty one % of patients with normal triglyceride levels had a 1 % or greater decrease in HbA1c.as contrasted to 27 % in those with elevated triglyceride levels. In both populations a substantial number of patients had a poorer response. This can only partially be explained by the baseline HbA1c level as shown in Fig. 2 where the correlation between baseline HbA1c and the decrease in HbA1c with DIAMOND treatment was 0.475, *p* < 0.0001 indicating that there are other patient factors influencing the glycemic response.

**Fig. 3:**
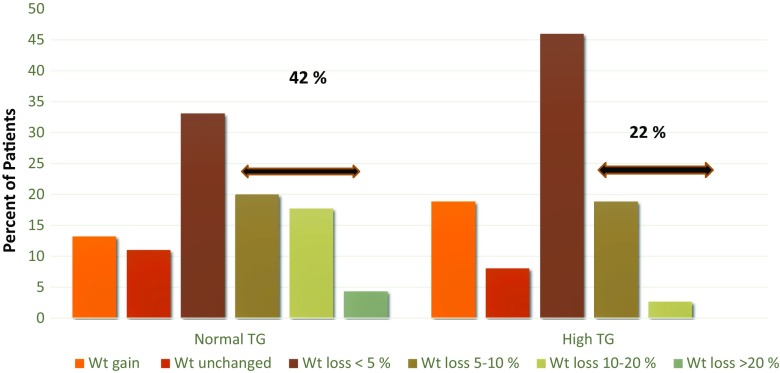
Frequency of weight change in patients with type 2 diabetes with normal (≤1.70 mmol/l) and high (> 1.7 mmol/l) fasting plasma triglyceride (TG) levels treated with meal-mediated glucose electrical stimulation for 12 months. Mean baseline weight for 45 normal TG patients 105 ± 5 kg and for 37 high TG patients 107.9 ± 3.6 kg. Forty two % of normal TG patients and 22 % of high TG patients lost ≥5 % of their body weight

The effect of DIAMOND therapy on weight loss in this same diabetic population is shown in Fig. 3. As noted with glycemic improvement, weight loss is greater in both magnitude and number of patients achieving 5 % or greater weight loss in 12 months. Weight loss occurs in the absence of a specific lifestyle intervention strategy as part of the clinical protocols, Table 3 which presents long-term data of a subset of patients with normal triglycerides many of whom had achieved significant weight loss. Most maintain their weight loss over periods as long as 3 to 6 years.

**Table 3:** Long-term treatment of 22 normal triglyceride patients with type 2 diabetes with the DIAMOND device: change in body weight

Patient	Mean TG (mmol/l)	Weight (kg)					Weight Loss (kg)			
		Baseline	Year 1	Year 2	Year 3	Year 5	Year 1	Year 2	Year 3	Year 5
1	1.44	151.1	115.0	118.0	122.0		−36.1	−33.0	−29.1	
2	1.20	97.3	87.0	83.0	86.0		−10.3	−14.3	−11.3	
3	1.06	95.6	80.0	83.0	88.0		−15.6	−12.6	−7.6	
4	1.47	114.0	101.0	96.0	102		−13.0	−18.0	−12.0	
5	1.15	123.0	111.0	104.0			−12.0	−19.0		
6	1.27	83.0	82.0	85.0			−1.0	2.0		
7	0.90	144.0	140.0	133.0			−4.0	−11.0		
8	1.51	80.0	66.0	74.0			−14.0	−6.0		
9	1.14	109.0	102.0	109.0			−7.0	0		
10	1.29	105.0	105.0	91.0			0	−14.0		
11	1.05	91.0	86.0	89.0			−5.0	−2.0		
12	1.63	75.0	71.5		73	65.0	−3.5		−2.5	−10.0
13	0.85	76.0	77.0		71.0	70.0	1.0		−5.0	−6.0
14	1.55	92.5	84.0	85.0		82.0	−8.5	−7.5		−10.5
15	1.14	76.0	72.0			73.0	−4.0			−3.0
16	1.19	83.0	72.0		71.0	72.0	−11.0		−12.0	−11.0
17	1.55	82.1		78	78	80.0		−4.1	−4.1	−2.1
18	1.56	87.0	86.8	85.0			−0.2	−2.0		
19	1.31	107.0	97.8	99.0			−9.2	−8.0		
20	1.34	83.0	73.8	75.0			−9.2	−8.0		
21	1.19	146.3		149.0			2.7			
22	0.82	86.0	76.1	73.0		−9.9	−13.0			

In summary, non-excitatory gastric electrical stimulation is an alternative therapy for obese patients with type 2 diabetes failing to reach their target glycemic goals with oral anti-hyperglycemic agents. The response to therapy is enhanced in patients with normal fasting plasma triglycerides and moderately elevated baseline HbA1c levels. Associated medical benefits are a sustained weight loss, a decrease in body fat and a reduction in systolic blood pressure. Side effects are those usually seen with laparoscopic surgery. Heartburn, nausea, vomiting and abdominal discomfort are rarely observed. A few instances of local irritation or an infection have occurred at the pocket of the pulse generator. Additional studies are needed to understand the DIAMOND’s interaction with the gut-brain pathways and the mechanism of the triglyceride interaction.

### Gastric stimulatory devices under development

The abiliti® system (IntraPace, Inc) consists of a lead with two electrodes, a transgastric sensor which detects food intake and a stimulating electrode placed over the vagus nerve at the lesser curvature. A stimulator sends a signal to the electrode when food intake is detected. The system monitors physical activity with a 3-D accelerometer and food intake by an intragastric food sensor which records daily intake. Patient and medical staff monitor the results and use the information to modify the patient’s behavior. Twelve month data in an open label study of 27 obese subjects (mean BMI 40.0 ± 5.7 kg/m^2^) showed a mean percent excess weight loss (EWL) of 49.3 ± 19.2 [27]. It is not possible from the design of the study to determine the effect of the electrical signal versus the effect of food and exercise monitoring on behavioral modifications. A second open label prospective study reported the results of 27 months of treatment in 32 obese subjects (mean BMI 42.1 ± 5.3 kg/m^2^). At 12 months the mean excess weight loss was 28.7 %. At 27 months the mean percent EWL was 22.5 % [28]. Eating behavior improved and weekly physical activity increased significantly (*p* < 0.001).

## Conclusions

Gastric electrical stimulation for effectively treating moderate (class 1 and class 2) obesity has remained elusive. The intimate neural and hormonal relationships between the stomach, the brain and the liver and pancreatic islets have suggested that modifying the neural pathways from the stomach to the regulatory centers for satiation and satiety in the hindbrain could be an effective means of reducing energy intake and decreasing body weight in obese individuals. The various devices reviewed use different locations, strategies and targets. Some provide a block of the vagal nerve trunks near the gastro esophageal junction; others target the antrum. Some provide stimulation for 12 or 24 h a day; others are only active postprandial. Some activate selective distal vagal nerve receptors that are nutrient selective and sensitive; others activate non-selective gastric neutral plexus.

Combining lifestyle modification with gastric electrical stimulation frequently complicates the interpretation of the results and requires that the studies be double blind and inactive implanted device controlled. An overlooked issue is that the constellation of causes of obesity are multiple and may differ among obese individuals, The heterogeneity of responses may provide clues that some forms of gastric electrical stimulation may effectively reduce body weight in some individuals and not others, The data presented in Fig. 2 and Table 3 showing that some patients lose ≥15 % of their body weight while others lose little or no weight supports this hypothesis. The relationship between the other metabolic benefits of gastric electrical stimulation in improving glycemic control and lowering systolic blood pressure are intriguing and need further study. Electrical stimuli control most physiologic processes and unraveling their potential effects in the gastric modulation of metabolism is still a challenge.
